# On the Microstructure, Microhardnessand Wear Behavior of Gray Cast Iron Surface Layer after Laser Strengthening

**DOI:** 10.3390/ma15031075

**Published:** 2022-01-29

**Authors:** Eugene Feldshtein, Oleg Devojno, Szymon Wojciechowski, Marharyta Kardapolava, Iryna Kasiakova

**Affiliations:** 1 Institute of Mechanical Engineering, University of Zielona Góra, Prof. Z. Szafrana 4, 65-516 Zielona Gora, Poland; 2 Faculty of Mechanical Engineering, Belarusian National Technical University, Khmelnitsky Str., 9, Build. 6, 220013 Minsk, Belarus; devoino-o@mail.ru (O.D.); margokardo@tut.by (M.K.); i.kosyakova88@gmail.com (I.K.); 3 Faculty of Mechanical Engineering, Poznan University of Technology, Piotrowo 3, 60-965 Poznan, Poland

**Keywords:** gray cast iron, laser strengthening, surface energy density, microstructure, microhardness, tribological behavior

## Abstract

Cast iron is one of the most common structural materials and is widely used in mechanical engineering production. Taking into account its rather low mechanical properties, different technologies are currently used in industry, among other areas, for the mechanical and thermal strengtheningof the surface layer, as well as surface alloying of workpieces. The aim of this study was a comprehensive analysis of changes in the microstructure, microhardness of the surface layer and its wear resistance under lubrication friction conditions and changed surface energy density in order to ensure the effectiveness of laser strengthening of gray cast iron. In this research, the efficiency of gray cast iron GJL200 laser strengthening was described. The basic properties of the surface layer of gray cast iron under laser strengthening, including the microstructure, microhardness, tribological and wear behavior, were compared with the properties of cast iron in the initial state. It was found that laser strengthening under the right choice of the surface energy density ensured a five-to-tenfold increase in the wear resistance of gray cast iron in comparison with the initial state. This was due to forming unconventional pseudo-vermicular graphite shapes at the friction zone, as well as a spongy-capillary effect appearance. The appropriate selection of surface energy density values provided stable and low coefficients of friction and a very significant increase in the wear resistance compared with the values reached for a cast iron in the initial state. This fact is new and very important for the engineering practice. The values of the surface energy density can be easily controlled, which means that different parts can be operated efficiently after laser strengthening.

## 1. Introduction

Cast iron is one of the most common structural materials, used first of all in series and mass production. Depending on the purpose, white, gray, ductile or high-strength cast irons may be used. Gray cast irons are characterized by good casting properties and are widely used in mechanical engineering. Their high brittleness makes it impossible to use them for parts working under tension or bending. Therefore, gray cast irons are used only for the parts working under compression. The industry uses technologies that increase the properties of ready-to-use molds from traditional grades of cast iron. Such technologies include mechanical and thermal strengthening of the surface layer, as well as surface alloying of work-pieces.

The effect of vibratory shot peening on the surface roughness and residual stresses of parts made of spheroid graphite cast iron was investigated in [1]. After processing, compressive residual stresses in the range of 170–330 MPa were formed in the surface layer. The effect of shot peening on the wear under dry sliding of as-cast and austempered ductile irons was studied in [2]. After processing, it was observed thatthe surface hardness of austempered samples increased due to martensite forming, whereas wear resistance decreased due to an increase in the surface roughness. The laser cavitation peening of HT200 gray cast iron was studied in [3]. It was possible to reduce the surface roughness while preserving the hardened layer. It was also shown that the surface roughness of the cast iron surface increased after processing, but the presence of the copper coverage layer reduced the surface roughness. The laser energy was optimized to provide the required roughness, compressive residual stress and microhardness. The surface melting process of nodular cast iron was investigated in [4]. The presence of retained austenite and martensite in most of the treated layers was confirmed. The wear of austempered gray cast iron and ductile iron to which Fe-Si-Mg andCa-Si additives were added was studied in [5]. It was shown that an additional heat treatment increased the dry friction coefficient, which is very important for friction pairs. The effect of surface melting and solidification on the structure and hardness of nodular cast iron was investigated in [6]. The laser beam and TIG method were used for surface melting. The research showed that surface melting resulted in complete dissolution of the graphite nodules and re-solidification of the dendritic structure, and the laser impact allowed for the formation of a more favorable microstructure. The microhardness of cast iron after additional melting increased significantly. The surface of nodular graphite cast iron melted using a CO_2_ laser was analyzed in [7]. Laser melting resulted in the formation of an inter-dendritic structure with a very fine ledeburite eutectic structure, good homogeneity, high hardness and dissolution of the graphite nodules. The erosion resistance of laser-treated iron was 110 times higher than that of the initial iron. Experimental studies of the solid-phase transformations during laser surface strengthening of cast iron were carried out in [8] in order to study the effect of processing conditions on the geometry, size and hardness of the heat-affected zone. It was possible under optimal processing conditions to provide an increase in microhardness at a considerable depth and the formation of micrometrical dimples, which can significantly improve the lubrication effect. The effect of laser surface treatment on the microstructure, crack resistance and stresses in the layers formed after laser remelting and strengthening of malleable irons was investigated in [9]. The hardened layers reached thicknesses from 1.5 to 2.5 mm. It was shown that it is possible to reduce thermal stresses and cracking intensity by regulating the energy density. It was shown in [10] that laser surface strengthening promoted the formation of martensite and carbide-based phases, which provided a hardness above 65 HRC and high abrasion resistance. Gray and nodular cast iron after surface remelting with a plasma beam were studied in [11], and it was shown that the melted surface had no pores and cracks and consisted of inter-dendrites and eutectics with a hyper-eutectic structure. The microhardness increased by 3–3.5 times. The fatigue wear resistance of gray cast iron after laser surface modification was studied in [12]. In this case, a surface consisting of soft and hard phases was formed. Laser treatment not only delayed the formation of microcracks but also significantly hindered their further propagation. A diagram of laser heat treatment, combining such technologies as remelting, alloying, strengthening from the solid state, tempering the surface layer of gray iron depending on the power density value and laser beam impact time were designed in [13]. The laser surface modification of GJS 400-12 ferritic ductile cast iron to improve tribological properties was used in [14]. The laser surface treatment resulted in a significant increase in the wear resistance of the cast iron due to an increase in the surface hardness; however, the friction coefficient increased as a result of an increase in both the adhesive and abrasive friction processes. The effect of laser treatment of GJS 400 and GJL 300 cast irons on the microstructure and dry sliding wear behavior was studied in [15]. Laser treatment increased the wear resistance of GJS 400 cast iron but, at the same time, resulted in an increase in the friction coefficient. The best performance was observed for a lower energy density due to a lower surface hardness and related superior toughness. The effect of quenchtempering and laser surface strengthening on the wear of gray cast iron was investigated in [16]. It was revealed that under laser treatment, the strengthening zone containing ledeburite with a hardness of about 68 HRC was formed. The heat-affected zone contained martensite with a hardness of about 66HRC and the substrate had a significantly reduced hardness. Under conditions of sliding wear, laser surface strengthening resulted in higher wear resistance compared with the initial material, but lower wear resistance than that of the austempered material. The effects of quench-tempering and laser-strengthening treatments on the wear resistance of gray cast iron were analyzed in [17] and the features of microstructure, microhardness and wear behavior of gray cast iron were described. The sliding wear behavior of the laser-hardened austempered ductile iron surface was described in [18].

In summary, the laser surface strengthening of cast iron parts showed its high efficiency, but it is used, first of all, for malleable cast irons. There are investigations of changes in their structure and mechanical properties, but the operational (tribological) properties have been studied only under conditions of dry abrasive friction. The purpose of this study was a comprehensive analysis of changes in the microstructure, microhardness of the surface layer and its wear resistance under lubrication friction conditions and changed surface energy density in order to ensure the effectiveness of the laser strengthening of gray cast iron.

## 2. Materials and Methods

### 2.1. The Initial Material

**Figure 1:**
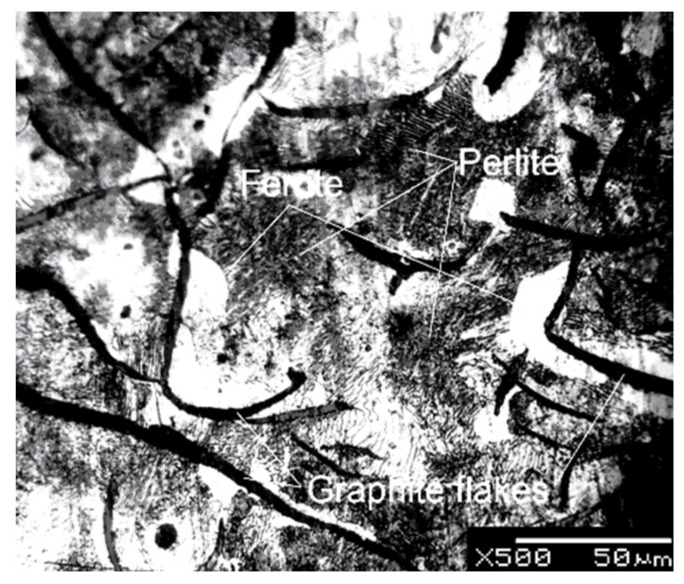
The microstructure of the initial GJL 200.

Gray cast iron EN-GJL200 was used as the initial material (Figure 1). This is pearlitic-ferritic cast iron, which was produced at OJSC “MTW” (Minsk, Belarus). According to EN 1561:1997, it contains 3.10–3.40% C, 1.90–2.30% Si, 0.60–0.90% Mn, ≤0.15% P and ≤0.15% S. Its mechanical properties were as follows:Brinell hardness 170–210, tensile strength 200 MPa, compressive strength 800 MPa and shear stress 230 MPa.

### 2.2. Laser-Strengthening Conditions

A Kometa CO_2_ continuous-action laser (TechnoLaser, Shatura, Russia) with a power of 1 kW was used for the laser processing. The analysis of the effect of the laser-processing conditions on changes in the surface layer properties was performed with laser beam diameters *d* of 1 and 2 mm and remelting speeds *v* = 100–600 mm/min. The overlapping ratio was equal to 1.0.

### 2.3. Tribological Testing

Friction and wear behavior were investigated under concentrated contact conditions according to the “roller–block” scheme. Samples (blocks) were made of cast irons and counter-bodies (rollers) were made of the AISI 1045 medium carbon steel. The counter-bodies hardness was 45–51 HRC. Samples were ground by electro-corundum (aluminum oxide) grinding wheels under a constant grinding condition in order to exclude the influence of the grinding process on the surface roughness of contact surfaces. Stable loads of 500 N and 1000 N were applied at a0.45 m/s sliding speed. The single test time was 1 h. L–AN 68 mineral machine oil (Jaśle Refinery, Jasło, Poland) was used as the lubrication medium and the oil drop-feed method was applied to lubricate the friction zone with a minute rate of 30 drops.

When testing, the momentary coefficient of friction (COF) *f* and temperature *T* in the friction zone were registered over time. Volumetric wear of the samples *V*_w_ and wear rate *I_V_* were determined based on the measured width of the wear traces. The following equations were used for the calculations:

(1) $$\mathrm{momentary}\;\mathrm{COF}\;f=\frac{2M}{FD_{r}},$$

where *M*—the friction moment, *F*—the load and *D*_r_—the roller diameter;

(2) $$\mathrm{volumetric}\;\mathrm{wear}\;V_{w}=\frac{D_{r}^{2}B}{8}\left\{2\arcsin\left(\frac{s}{D_{r}}\right)-\sin\left[2\arcsin\left(\frac{s}{D_{r}}\right)\right]\right\},\;{\mathrm{mm}}^{3},$$

where *B*—the width of sample and *s*—the average width of wear groove;

(3) $$\mathrm{wear}\;\mathrm{rate}\;I_{V}=\frac{V_{w}}{L},\;{\mathrm{mm}}^{3}/\mathrm{km},$$

where *L*—the friction path.

### 2.4. Measuring Equipment

A Buehler Micromet 2 tester (Spectrographic Ltd., Leeds, UK) was used to control the microhardness of the samples and the weight of 100 g was applied. The surface roughness parameters were measured with a TR-200 tester (Salu Tron GmbH, Frechen, Germany). The XRD method was used to analyze the phases in remelted and solidified layers. Tests were performed using a DRON 3.0 device (Bourevestnik JSC, Saint Petersburg, Russia) with Cu-monochromatic radiation at arotation speed of the sample equal to 1 deg/min in a 10–75° range of angles. Under the conditions used, the layer thickness with 75% absorption of the primary beam energy was equal to 3–12 μm. The results of the XRD analysis were processed using the ARSANAL software. Features of the microstructures were studied with a metallographic system MI-1 with an inverted microscope and SIAMS 800 analyzer (Optoelectronic Systems JSC, Minsk, Belarus). Worn surfaces were analyzed using a scanning electron microscope JSM-5600LV (JEOL Ltd., Tokyo, Japan).

Tribological tests were performed using a self-made A-135 friction tester. The values of friction moment and friction time were registered usingsuitable sensors and the values of temperature in the friction zone were measured usinga chromel–alumel thermocouple. A DinoLite digital microscope (ANMO Electronics Corporation, New Taipei City, Taiwan) was used for measuring the samples’ wear with an accuracy of 0.001 mm.

Statistical processing of the results was carried out using the Statistica 13 software package. In order to validate the results, the tests were re-run three times at each measuring point and the standard error calculated was equal to 5%.

## 3. Results

### 3.1. The Surface Roughness Changes

**Figure 2:**
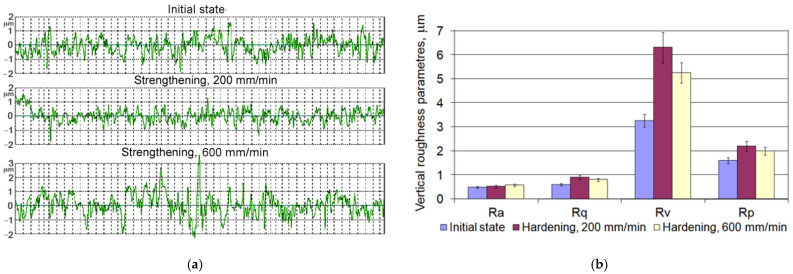
Surface roughness profiles (**a**) and values of the surface roughness parameters (**b**) in the initial state and after the laser strengthening with *d* = 1 mm, *v* = 200 mm/min and *d* = 2 mm, *v* = 600 mm/min.

The analysis of the examined surfaces of the samples showed that the microroughness profiles after grinding the cast iron in the initial state and after laser strengthening differed (Figure 2). After the laser strengthening, stand-alone high protrusions and deep valleys were observed that were probably caused by the influence of the laser beam on the graphite flakes and their pitting. Changes in technological parameters of laser strengthening had a small influence on the surface roughness parameters.

### 3.2. The Microstructure of Strengthened Layers

**Figure 3:**
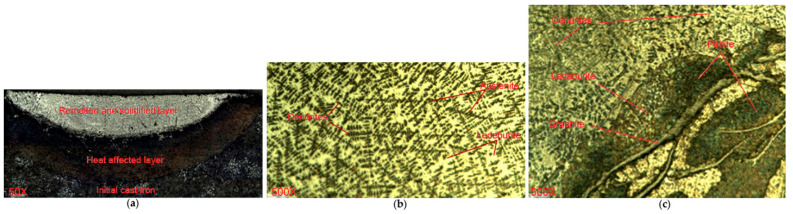
Microstructures of cast iron in the laser beam effect area: (**a**) general view, (**b**) remelted and solidified zone and (**c**) solid-state strengthening zone.

When processing cast irons usinga laser beam with melting of the surface layer, the laser-affected zone is not homogeneous in depth and has a layered structure (Figure 3a). The upper layer (remelted zone with subsequent solidification) is formed during quenching from the molten state. In this layer, graphite dissolves in the molten pool and, after cooling and solidification, the structure that is formed consists of small dendrites of austenite that have grown during crystallization of the molten metal and are surrounded by dispersed ledeburite. The dendrites usually have first- and second-order axes, less often third-order axes, since there is not enough time for their formation.

Crystallization was finished with the formation of a columnar cell-dendrite structure, with the dendrites extended in the direction of heat removal (Figure 3b). The microstructure of the upper layer was practically independent of the laser treatment parameters. The second layer (solid-state strengthening zone) was formed during cooling with quenching. Its lower boundary was defined by heating to the temperature of the *A_c_*_1_ point, and both complete and incomplete quenching took place in this layer. This layer was characterized by a significant non-uniformity of the structure along the depth. Closer to the alloyedsurface, due to high cooling rates, lamellar ledeburite was formed from the zone of homogeneous austenite. However, in areas closer to the initial metal structures, the lamellar ledeburites were formed out of the zone of non-homogeneous austenite during cooling (Figure 3c). The third layer (transition zone) was formed when heating the metal below the *A_c_*_1_ temperature point. In this layer, the formation of troostite or sorbite took place. The second and third layers formed a heat-affected zone (HAZ).

In the upper section of the HAZ, the cast iron matrix around the graphite was melted and saturated with carbon. In this section, the following structural components were formed: a thin area with ferrite predominance near the graphite flakes, then a lamellar ledeburite, a mixture of ledeburite and austenite and, finally, a homogeneous area of ferrite and pearlite. Increasing the laser spot speed reduced the surface energy density and caused a decrease in the degree of carbon saturation of the iron matrix around the graphite inclusions. In the lower part of the HAZ, the incompleteness of austenitization increased;therefore, the basic solid solution was saturated with carbon to a lesser degree.

**Figure 4:**
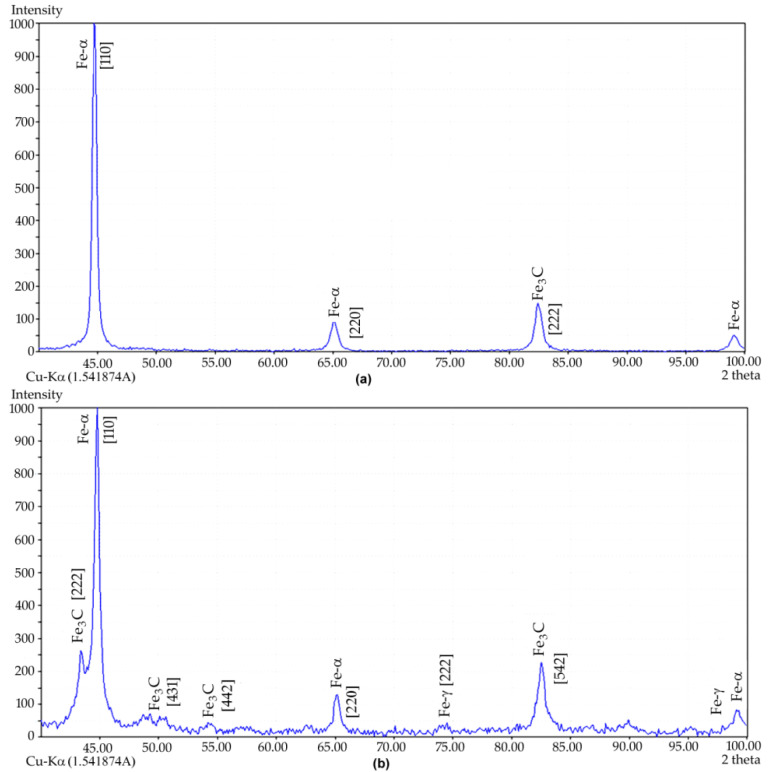
XRD diagrams for cast iron: (**a**) in the initial state and (**b**) under laser strengthening.

**Figure 5:**
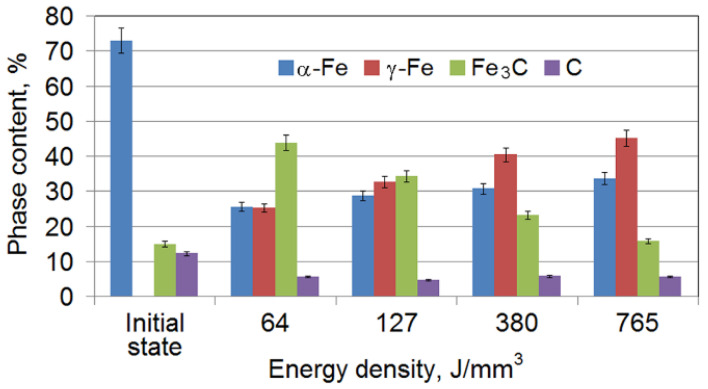
The content of the structural components in cast iron as a function of energy density when laser strengthening was applied.

The XRD analysis of cast iron samples revealed characteristic changes in the content of the main structural components after laser treatment (Figure 4). As the surface energy density increased, the austenite γ-Fe content in the cast iron increased, the amount of cementite Fe_3_C decreased and the graphite content fluctuated between 5.7 and 4.6% (Figure 5). It should be added that austenite was absent in the initial cast iron.

### 3.3. Changes in Strengthened Layer Thickness after Laser Strengthening of Cast Iron

**Figure 6:**
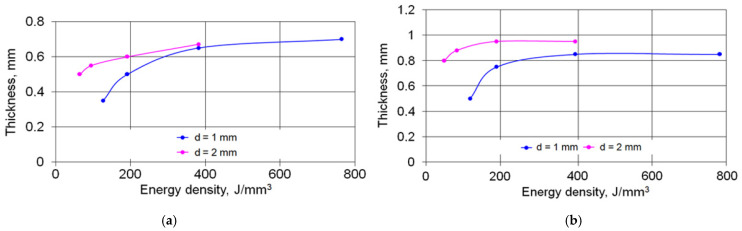
The effect of laser beam energy density: (**a**) on the thickness of the melted–solidified layer and **b**) on the thickness of the HAZ.

The thickness of the hardened layer, as well as the thickness of the HAZ, depended on the surface energy density of the laser beam, which decreased with increasing the spot diameter and speed. For the remelted–solidified layer, the yield of the thickness values on the plateau was observed when the surface energy density reached 600 J/mm^3^, whereas, for the HAZ, it occurred when the energy density was above 200 J/mm^3^ (Figure 6). This character of changes was related to the conditions of heat removal and the solidification rate of the molten laser pool.

### 3.4. Changes in the Microhardness of the Strengthened Surface Layer

**Figure 7:**
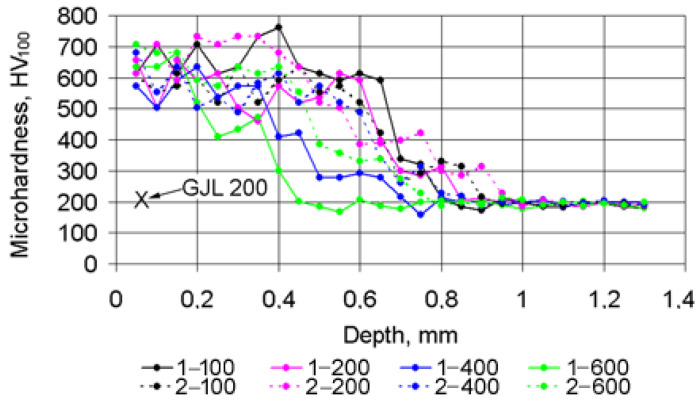
Microhardness distribution along the depth after the laser strengthening (used designations: digits 1 and 2—beam diameters 1 and 2 mm; digits 100, 200, 400 and 600—beam speeds 100–600 mm/min; X—GJL 200 in the initial state).

Laser strengthening of the GJL 200 cast iron significantly affected the microhardness of the surface layer, leading to an increase in microhardness (Figure 7). Depending on the parameters of the laser processing, the microhardness of hardened cast iron increased 2–3.5 times in comparison with the microhardness HV_100_ = 190–230 of the initial cast iron.

**Figure 8:**
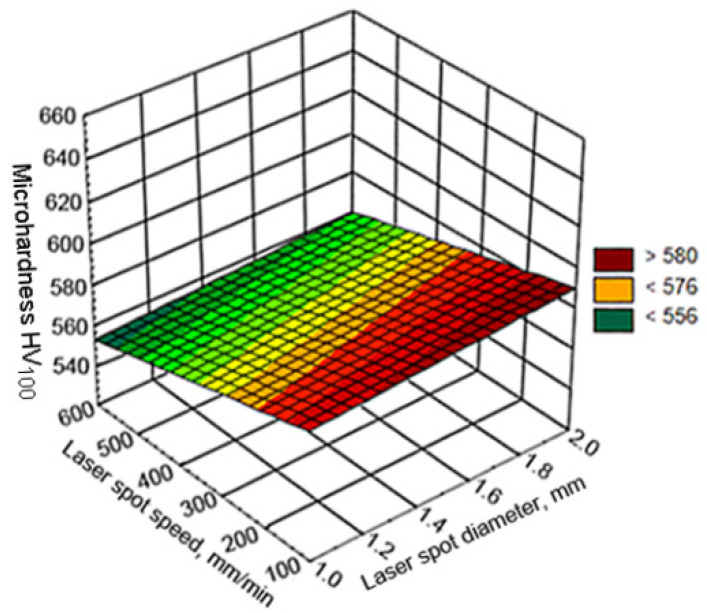
Changes in hardness as a function of the laser spot speed and diameter.

The effects of the laser strengthening parameters on the microhardness of the remelted and solidified layer were insignificant (Figure 8) and can be described by the dependence HV_100_ = 581.4 + 7.25*d* − 0.06*v*.

### 3.5. Tribological Behavior of the Cast Iron Surface Layer after Laser Strengthening

**Figure 9:**
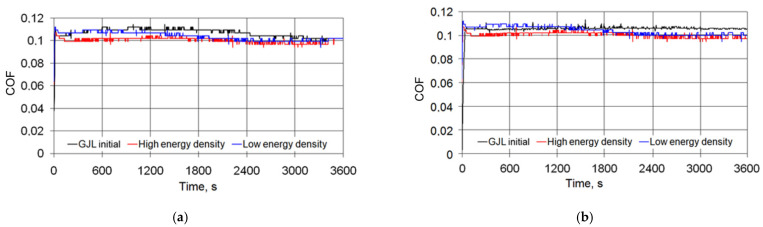
Changes over time of momentary COFs after gray cast iron laser strengthening: (**a**) under a 500 N load and(**b**) under a 1000 N load.

An analysis of changes over time of momentary COFs of gray cast iron samples after laser strengthening revealed that under relatively low loads, the values of the COFs were practically constant, i.e., laser treatment conditions, as well as friction time, had no impact (Figure 9a). With increasing load, the COFs changed, and after the running-in time, their value after laser strengthening decreased by 20–30% in comparison with the initial cast iron and stabilizedover time (Figure 9b).

**Figure 10:**
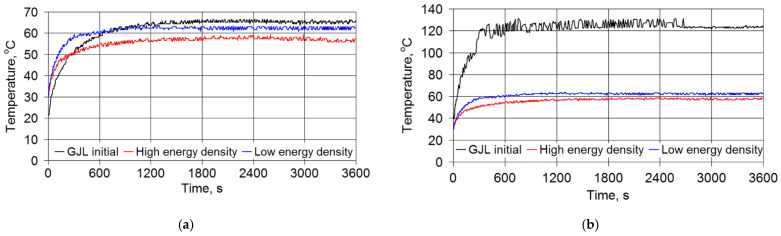
Changes in friction temperature versus time after gray cast iron laser strengthening: (**a**) under a 500 N load and (**b**) under a 1000 N load.

The temperatures in the friction zone depended on the load level. If the loads were negligible, the temperature after the running-in time did not depend on the wear time or the laser strengthening conditions (Figure 10a).

Laser strengthening provided an advantage over the initial cast iron, both regardingthe stability and temperature level. Under high loads (Figure 10b), the temperature at the friction zone of the initial cast iron reached 120–140 °C, while in the case when laser strengthening was applied, its level did not change or even slightly decreased. The feature described was due to the change in the surface energy density of the laser beam.

**Figure 11:**
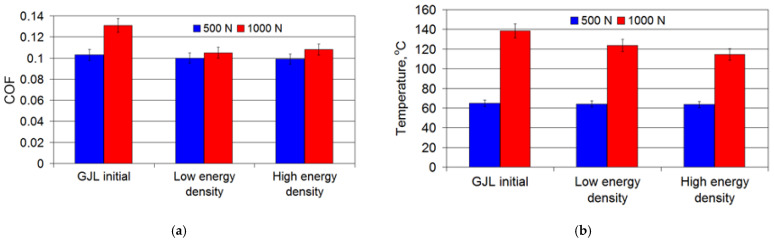
Effect of the laser treatment conditions on (**a**) the COF and (**b**) the temperature in the friction zone.

The intensity of the effect of the laser treatment parameters on the tribological behavior of cast irons is shown in Figure 11. The high energy density of the laser beam reduced the COF and the temperature in the friction zone in comparison with the initial cast iron and laser treatment under the low energy density.

### 3.6. Wear Features of the Surface Layer of the Cast Iron after Laser Strengthening

**Figure 12:**
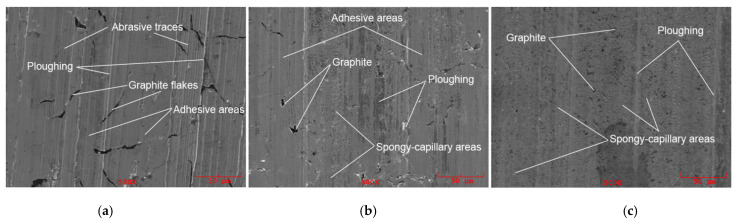
Worn surfaces of samples made of (**a**) initial cast iron, (**b**) strengthened cast iron under the middle energy density of the laser beam and (**c**) under the high energy density of the laser beam.

SEM studies of the worn surfaces of the cast iron specimens after laser strengthening were performed after the friction cycle under different surface energy densities and a load of 1000 N because the differences in the wear character were more noticeable with significant loads. The worn surface of the cast iron samples had a structure similar to that of cast iron in the initial state (Figure 12a). Traces of abrasion, as well as plowing and adhesion areas, were observed there. The worn surfaces of the samples in the hardened state changed significantly.

Under laser strengthening and with the action of significant loads and high temperatures, the graphite particles were crushed and changed their shape from flake to pseudo-vermicular (Figure 12b). According to [19], cast iron with a vermicular structure is characterized by a significantly greater wear resistance compared with conventional gray cast iron. It was also found that changes in the surface layer microstructure promote the formation of spongy-capillary areas, which were noticeable on the wear surfaces, especially at a high surface energy density (Figure 12c), and which provided higher wear resistance. A similar effect was described in [20].

The spongy-capillary effect promoted the formation of a tribofilm on the worn surfaces, improving the friction conditions, as described above. Its presence was confirmed by the relatively high content of phosphorus and sulfur on the friction surfaces, which were part of the machine oil used. Their content was 3–4 times higher compared with the worn surface of the base cast iron.

**Figure 13:**
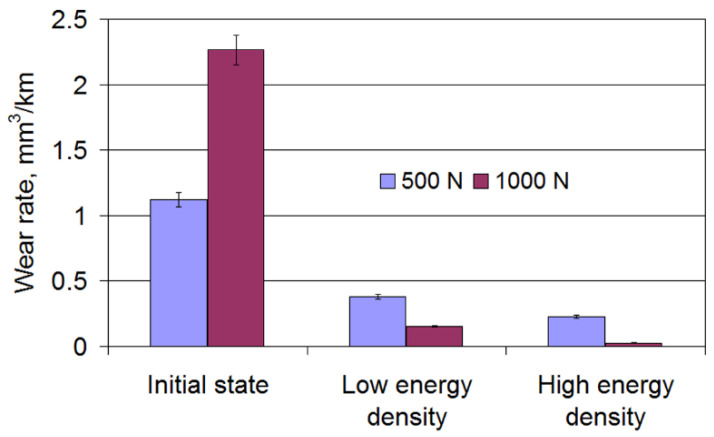
The effect of the laser beam energy density on the wear rate of cast iron under laser strengthening.

Changes in the wear rate of the surface layer of cast iron after laser strengthening are shown in Figure 13. The registered character of changes was caused by changes in the microhardness and microstructure of the surface layer, depending on the surface energy density of the laser beam. The right choice of strengthening conditions allowed for increasing the wear resistance of the cast iron after strengthening from fivefold to almost tenfold in comparison with the initial cast iron.

## 4. Discussion

In this study, the efficiency of laser strengthening of gray cast iron was investigated. The effect of the laser processing conditions was studied with different laser spot diameters and speeds and changes in the base features of the strengthened surface layer were found, primarily the structures, microhardness and friction and wear behavior. The surface layer microstructures were not homogeneous in depth and had a layered texture. The upper layer consisted of small first- and second-order axis dendrites that grew during the crystallization of the molten metal and thatwere surrounded by dispersed ledeburite. The microstructure of the upper layer was practically independent of the laser treatment parameters. Below the abovementioned layer, the structure was characterized by significant non-uniformity and the following structural components were formed there: a thin area with ferrite predominance near the graphite flakes, then a lamellar ledeburite, a mixture of ledeburite and austenite and, finally, a homogeneous area of ferrite and pearlite.

Laser strengthening significantly affectedthe microhardness of the surface layer: it increased 2–3.5 times in comparison with the microhardness of the initial cast iron.

Under low loads, the worn surface of the cast iron samples had a surface topography similar to that of the cast iron in the initial state. However, under the action of significant loads and high temperatures, the graphite particles were crushed and changed their shape from flake to pseudo-vermicular. Changes in the surface layer microstructure promoted the formation of spongy-capillary areas, which were noticeable on the wear surfaces. This effect promoted the formation of a tribofilm on the worn surfaces, improving the friction conditions.

Grey cast iron continues to be one of the most widely used structural materials and the technologies for improving its operating properties are constantly evolving, as evidenced by the publications presented above. The vast majority of them considered the basic indicators of laser processing, namely, laser spot speed and diameter. Research revealed that the use of a more universal indicator, namely, an energy density, allowed for the successful selection of more effective processing conditions, and such data for the widely used GJL 200 gray cast iron werealready introduced. Studies were realized with a wide range of values of surface energy density and it was revealed that its best choice provided a significant increasein the surface layer wear resistance.

## 5. Conclusions

Based on the research results analysis, the following was found:

1. When processing cast irons with a laser beam with remelting and solidification of the surface layer, their microstructure was not homogeneous in depth and had a layered texture. The thickness of the strengthened layer, as well as the thickness of the HAZ, depended on the surface energy density of the laser beam, which decreased with increasing spot diameter and speed. For the remelted–solidified layer, the yield of the thickness values on the plateau was observed when the surface energy density reached 600 J/mm^3^, whereas for the HAZ, it occurred when the energy density was above 200 J/mm^3^.
2. The effects of laser strengthening parameters on the microhardness of the remelted and solidified layer were insignificant.
3. Under relatively low loads, the COFs after laser strengthening were practically constant, and with increasing loads, the COFs decreased by 20–30% in comparison with the initial cast iron. Under high loads, the temperature of the initial cast iron reached 120–140 °C, while in the case of laser strengthening, its level was considerably lower. Laser strengthening allowed for increasing the wear resistance of cast iron from fivefold to almost tenfold in comparison with the initial cast iron.
4. Most important for the engineering practice was the fact that when laser strengthening was applied to gray cast iron, the right choice of the surface energy density provided stable and low COFs and a very significant increase in the wear resistance compared with the cast iron in the initial state.
5. The right choice of the surface energy density values provided stable and low coefficients of friction and a very significant increase in the wear resistance compared withthe cast iron in the initial state. The values of the surface energy density could be easily controlled, which meant that different parts could be operated efficiently after the laser strengthening.

## Data Availability

The data presented in this study are available on request from the corresponding author.

## References

[1] Zaleski K., Skoczylas A. (2019). The influence of vibratory shot peening on the selected properties of the surface layer elements made of cast iron. Mechanik.

[2] Silva K.H.S., Carneiro J.R., Coelho R.S., Pinto H., Brito P. (2019). Influence of shot peening on residual stresses and tribological behavior of cast and austempered ductile iron. Wear.

[3] Gu J., Luo C., Zhang P., Ma P., Ren X. (2020). Laser cavitation peening of gray cast iron: Effect of coverage layer on the surface integrity. Appl. Surf. Sci..

[4] Abboud J.H., Benyounis K.Y., Olabi A.G., Hashmi M.S.J. (2007). Laser surface treatments of iron-based substrates for automotive application. J. Mater. Process. Technol..

[5] Akinribide O.J., Akinwamide S.O., Obadele B.A., Ogundare O.D., Ayeleru O.O., Olubambi P.A. (2021). Tribological behaviour of ductile and austempered grey cast iron under dry environment. Mater. Today Proc..

[6] Benyounis K.Y., Fakron O.M.A., Abboud J.H., Olabi A.G., Hashmi M.J.S. (2005). Surface melting of nodular cast iron by Nd-YAG laser and TIG. J. Mater. Process. Technol..

[7] Alabeedi K.F., Abboud J.H., Benyounis K.Y. (2009). Microstructure and erosion resistance enhancement of nodular cast iron by laser melting. Wear.

[8] Bhavikatti S.S., Pardeshi S.S., Mishra P.K. (2012). Investigation for Hardening of Cast Iron using Low-Power Fiber Laser. Int. J. Eng. Innov. Technol..

[9] Fernandez-Vicente A., Pellizzari M., Arias J.L. (2012). Feasibility of laser surface treatment of pearlitic and bainitic ductile irons for hot rolls. J. Mater. Process. Technol..

[10] Němeček S. (2013). Microstructure and properties of cast iron after laser surface hardening. Mater. Eng..

[11] Cheng X., Hu S., Song W., Xiong X. (2014). A comparative study on gray and nodular cast irons surface melted by plasma beam. Vacuum.

[12] Chen Z., Zhou T., Zhang P., Zhang H., Yang W., Zhou H., Ren L. (2015). Influences of single laser tracks’ space on the rolling fatigue contact of gray cast iron. Opt. Laser Technol..

[13] Paczkowska M. (2016). The evaluation of the influence of laser treatment parameters on the type of thermal effects in the surface layer microstructure of gray irons. Opt. Laser Technol..

[14] Pagano N., Angelini V., Ceschini L., Campana G. (2016). Laser remelting for enhancing tribological performances of a ductile iron. Procedia CIRP.

[15] Ceschini L., Campana G., Pagano N., Angelini V. (2016). Effect of laser surface treatment on the dry sliding behaviour of the ENGJS400-12 ductile cast iron. Tribol. Int..

[16] Wang B., Pan Y., Liu Y., Barber G.C., Qiu F., Hu M. (2020). Wear behavior of composite strengthened gray cast iron by austempering and laser hardening treatment. J. Mater. Res. Technol..

[17] Wang B., Pan Y., Liu Y., LyuNBarber G.C., Wang R., Cui W., Qiu F., Hu M. (2020). Effects of quench-tempering and laser hardening treatment on wear resistance of gray cast iron. J. Mater. Res. Technol..

[18] Han X., Zhang Z., Pan Y., Barber G.C., Yang H., Qiu F. (2020). Sliding wear behavior of laser surface hardened austempered ductile iron. J. Mater. Res. Technol..

[19] Zhang M.X., Pang J.C., Qiu Y., Li S.X., Wang M., Zhang Z.F. (2020). Influence of microstructure on the thermo-mechanical fatigue behavior and life of vermicular graphite cast irons. Mater. Sci. Eng. A.

[20] Feldshtein E.E., Dyachkova L.N. (2020). Wear minimization for highly loaded iron-based MMCs due to the formation of spongy-capillary texture on the friction surface. Wear.

